# Stroke in young adults: about 128 cases

**DOI:** 10.11604/pamj.2014.17.37.3226

**Published:** 2014-01-20

**Authors:** Mohamed Chraa, Nesrine Louhab, Najib Kissani

**Affiliations:** 1 Neurology Department, Mohamed VI universitary hospital, Marrakesh, Morocco

**Keywords:** Ischemic stroke, young adults, risk factors, prognosis, prevention

## Abstract

Ischemic stroke is rare in young adults, but it is genuinely a serious situation giving the fact that it touch a very active part of our society. We report a series of 128 cases. The purpose is to analyze the risk factors, etiologies and outcomes of ischemic stroke in young adults in Marrakesh. Retrospective study performed at the Neurology department Mohammed VI universitary hospital in Marrakesh interesting 128 patients. The diagnosis of ischemic stroke was assessed through clinical and radiological confrontation. Results: The age of our patients varied from 18 to 45 years old, 76 males and 52 females giving a male: female ratio of 1:46. Hypertension was the first risk factor involved with 63 (49.2%) cases, followed by smoking with 52 (40.6%) patients. The causes of ischemic stroke were characterized by the predominance of the cardio embolic origin with 43 (33.5%) cases, the existence of 14 (11%) cases of syphilitic arthritis, and the 52 (40.6%) cases of unknown etiologies. The authors stress the difficulties faced on supporting ischemic stroke in southern Morocco in particular when concerned by the etiological finding and the rehabilitation after the acute phase of the stroke. Our study points out the high incidence of embolic heart disease in our context. The lack of neurologists, low coverage of the population and the underestimation by physicians are factors that can explain why ischemic stroke remain undiagnosed.

## Introduction

Ischemic stroke in young adults is considered as a relatively rare event, with fewer than 5% of all cerebral ischemic infarctions, it occurs on a population between 18 and 45 years old [1]. As expected, even if not as frequent as it is in elderly, stroke in young adults require an increasing interest given the fact that it interests an active part of the society, thereby the direct and indirect costs are astonishing, this is more palpable in our developing countries where the young adults are the mainstay of the economy, furthermore, it is a great occasion for physicians to study the physiopathology of stroke [2–6]. The etiologies of stroke among this population are various and require a full and thorough investigation. In developed countries, the mean etiologies are cervical artery dissection and atherosclerosis, when embolic cardiopathies are more incriminated in developing [4, 5]. The objectives of the present study were to evaluate the risk factors, etiologies and the prognosis of this disease in young patients in Marrakesh, knowing that such a studies are rarely conduct in our country.

## Methods

We performed a retrospective study over 4 years from January 2007 and December 2010, all patients between the ages 18 and 45 years (which define young adults in our study) admitted for ischemic stroke were included. The study was conducted in the department of neurology in Mohammed VI universitary hospital, in Marrakesh. Our hospital is the biggest in the region but patient from other towns may be referred to first or second level near their hometowns. Furthermore, in Marrakesh wealthy patients may be admitted to private clinics. Our city has a population which exceed 1000000. In another hand, our department is a unit of 22 beds, it accepts patients from the city of Marrakesh, its closer area, but also all the south of Morocco.

Information extracted from the folders included age, sex, occupation, time for initial admission, clinical features, presence or absence of risk factors such as hypertension, diabetes mellitus, hypercholesterolemia, history of cigarette smoking, contraceptive intake treatment, alcohol intake, sexual behaviour, a cardiac pre-existing disease (regarding this risk factors, they were retained in the bases of history regardless of the numerical values found at admittance), history of stroke in the patient or his closer parents, and the results of the follow up.

Hypertension (HT) was identified when the patient was previously diagnosed with HT by a clinician or had a systolic blood pressure of ≥140 mm Hg and/or a diastolic blood pressure of ≥90 mm Hg based on the average of 2 or more properly measured blood pressure readings. Diabetes mellitus was diagnosed according to the World Health Organization criteria (7). Hyperlipidemia was considered a risk factor when the fasting serum cholesterol level was ≥200 mg/dL and/or the serum triglyceride level was ≥150 mg/dL. All the patients performed a radiological investigation before their admission, in almost all the cases, the first exam to be conducted is the CT scan, in some cases the MRI was first performed, any way, the diagnosis of ischemic was certain in all the cases based on clinical and radiological statement.

All the patients performed a cardiovascular statement including chest X-ray, an electrocardiogram, an echocardiography and a Doppler of cervical arteries, and a full biological workup including meanly: complete blood count, fasting blood glucose, lipid profile, antibody to human immunodeficiency virus (anti-HIV), and Treponema pallidum hemagglutination (TPHA). Protein C, protein S, antithrombin III, fasting homocysteine, lupus anticoagulant, anticardiolipin, antinuclear antibodies, and erythrocyte sedimentary rate were measured within 1 week after onset of stroke in patients in whom no cause of stroke was yet identified after the first biological and cardiovascular workup. Patients with low levels of protein C, protein S, and antithrombin III were rechecked approximately 10 weeks later for confirmation before the instauration of any long term treatment.

Finally the residual deficit was established according to the modified Rankin Scale (mRS).

## Results

A total of 442 stroke patients were admitted during the 4 years period. Of this number, 128 (28.9%) were between 18 and 45 years old. The minimum age was 18 years, and the maximum age was 44 years with a mean age of 28.3 ± 4.2 years. There were 76 males and 52 females giving a male: female ratio of 1:46. The patients were from urban area in 77 cases (60% of all patients). The age and sex distribution of the patient are shown in Table 1.

**Table 1:** The age and sex distribution of our patients

Age Group	Male	Female	Total (%)
18-25	15	8	23 (18%)
26-35	22	19	41 (32%)
36-45	39	25	64 (50%)

The frequency of risk factors among the patients is summarized in Table 2. Hypertension was responsible for 63 (49.2%) cases, smoking was present in 52 (40.6%) patients, oral contraception intake was verified present in 40 women, pre-existing heart disease were reported in 23 (17.9%) cases, diabetes mellitus was present in 17 (13.2%) cases, excessive alcohol intake 11 (8%) cases, hyperlipidimia was reported in 10 (7.8%) cases, three patients had have TIA, two patients were migrinous and finally two patients presented their stroke during the postpartum period.

**Table 2:** Risk factors found in our patients

	Male	Female
Hypertension	36	27
Cigarette smoking	44	8
Oral Contraceptives	-	40
Heart Disease	11	12
Diabetes Mellitus	8	9
Heavy alcohol consumption	10	1
Hypercholesterolemia	4	6
TIA	2	1
Migraine	-	2
Post-partum period	-	2

The clinical presentation is shown in Table 3. The mean presentation was an hemiplegia or an hemiparesis in 103 (80.4%) cases, 44 (34.3%) patients had consciousness disorders going from confusion to coma, 69 (53.9%) patients has speech troubles, qualified as dysarthria in 33 (25.7%) cases and as aphasia in 36 (28.1%) cases, 21 (16.4%) patients had early onset epileptic seizures, 7 (5.4%) patients had a visual field lost, 3 (2.3%) patients had a brainstem syndrome.

**Table 3:** The clinical presentation

	Male	Female
Hemiperisis/hemiplegia	56	47
Consciousness disorders	23	21
Speech troubles	44	25
Dysarthria	20	13
Aphasia	24	12
Early onset epileptic seizures	11	10
Visual field lost	4	3
Brainstem syndrome	3	0

The radiological findings were as following: normal in 33 cases, and showing signs suggestive of a cerebral infraction in 95 (74%) cases, of this last category, we had a middle artery involvement in 83 (87.3%) cases, an anterior artery involvement in 3 (3.1%) cases, and a vertebrobasillary involvement in 9 (9.4%) cases.

The stroke etiologies are detailed in Table 4 the main findings are the predominance of the cardioembolic origin with 43 (33.5%) cases, dominated by valvular rhumatic diseases, the existence of 14 (11%) cases of syphilitic arthritis, and the 52 (40.6%) cases of unknown etiologies.

**Table 4:** The etiologies of stroke

	Male	Female	Total
Cardioembolic origin	22	21	43
Atherosclerosis	8	7	15
Cervical dissection	2	1	3
Syphilis	10	1	11
Prot S, c deficiency	1	1	2
Antiphospholipid syndrome	0	1	1
Sneddon's syndrome	0	1	1
Unkown etiology	34	18	52

The immediate evolution is characterised by the death of 21 (16.4%) patients, the follow up now ranges from 3 months to 82 months. The outcome is shown in Table 5, indeed, 49 (38.2%) recovered without any complication, 38 (29.6%) presented a residual motor deficit which is detailed in Table 6 following the modified Rankin Scale, 7 presented an epilepsy, and 2 patients suffer from a vascular dementia, finally 11 patients were lost to follow up.

**Table 5:** Outcomes of our patients

	Male	Female	Total
Full recovery	30	19	49
Residuel motor deficit	20	18	38
Epilepsy	5	2	7
Vascular dementia	1	1	2
Lost to follow-up	9	2	11
Death	14	7	21

**Table 6:** Residual motor deficit assessment function on the modified Rankin Scale

Level	Male	Female	Total
0	30	19	49
1	3	2	5
2	3	2	5
3	5	7	12
4	7	8	15
5	0	1	1
6	14	7	21

## Discussion

The incidence rates in the present study are representative of hospital inpatients, but in spite of all the bias of such a study, important information can be gathered; stroke in young adults are a relatively frequent situation in Marrakesh when compared to similar studies [1–3], this situation can be explain by the fact that etiological factors such as embolic cardiapathies and sexually transmitted infections (syphilis..) are frequent, also by the predominance of the young population in our society.

Our study found a male predominance with a male to female ration of 1.46, this result is consistent with the general finding of previous studies that found ischemic stroke to be more frequent in men until the age of 85 years where it becomes predominant in women [4–7]. Hypertension is widely considered as a major risk factor of stroke in the general population [8–12]. Chong and Sacco et Al, found in their study about race/ethnic differences in stroke among young adults that hypertension was more frequent in black compared to whites, suggesting a higher risk in African population [13], this was confirmed by another study performed by a Nigerian team who found hypertension to be responsive of stroke in 77.8% of all cases collected [14], our study found also hypertension to be the first risk factor in the rate of 49.2%, this is another proof of the major role of hypertension in the physiopathology of stroke among young people as it is for elderly. Cigarette smoking in young people is an important data because of the increasing prevalence of this behaviour in this age group [15, 16], and is highly suspected to increase the relative risk of having a stroke [17], in our study it is indeed a major risk found in 40.6% of all cases. The relationship between diabetes mellitus and stroke has been well documented in general populations [18–21], but not yet in young adults. The studies performed to analyse the effect of diabetes in the formation of an ischemic stroke didn't bring a real scientific proof to its involvement but it is indeed a highly suspected risk factor [22–24], our study found diabetes to be responsive of 13% of all cases, finally; approximatively 18% of our patients had a pre-existing heart disease, this situation is in agreement with the findings of general population studies that suggest embolic cardiopathies to be a high risk factor of stroke [25, 26]. The conclusions and recommendation that we should perform in order to improve the prevention of stroke among the young adults are; a more efficient and an early diagnosis of all this risk factors by the information and education of our population who is still very far from establishing the relationship between this factors and stroke.

The topography of cerebral infraction was dominated by the territory of the middle cerebral artery; this is consistent with other previous studies [27, 28]. The subtypes of stroke is variable depending on the degree of development of the country; the first suspected etiology in developed countries is cervical dissection, while heart diseases are predominantly incriminated in developing ones [29–31], in another hand the frequency of unknown etiology depends also on the quality and the level of equipments of the departments taking care of this pathology, though, the higher rates should be found in the developing countries, in our study almost 41% of all strokes remained of an unknown cause, this high rate can be explained by the limitations we found to perform all the necessary explorations (especially trans-oesophageal ecography, angio-scan, angio-MRI and other blood tests) which are out of our patients financial means, but we think. Stroke subtypes found in different studies are summarized in Table 7 [32–38].

**Table 7:** Comparison between the etiologies of ischemic stroke in young adults found in deferent studies

Year	Country	Age range	Sample	Stroke subtype (%)				
				LAA	SVO	CE	ODE	UE
1994	Saudi Arabia	15- 45	70	12.9	24.3	17.1	30.0	15.7
1998	USA	15- 44	428	3.8	19.8	31.1	13.3	32
2000	South Korea	15- 45	149	20.8	17.4	18.1	26.8	16.8
2002	Taiwan	15- 45	241	7.9	22.4	19.5	24.5	25.7
2006	Iran	15- 45	124	9.7*		54	8.1	28.2
2007	Qatar	15- 45	40	–	42.5	–	5	42.5
2007	Spain	15- 45	272	–	21	17	26	36
2007- 2010	Our study	18- 45	128	11,7		33.5	14,2	40.6

LAA = Large artery atherosclerosis; SVO = Small-vessel occlusion; CE = Cardiac embolism; ODE Other determined aetiology; UE = Undetermined aetiology

Death concerned 21 patients (16%), although it is a high level, in our opinion it is very encouraging knowing that we admit in our unit patients that must be hospitalized in intensive care units giving the lack of this last in our hospital.

The authors would like at last to stress the difficulties faced on supporting ischemic stroke in southern Morocco in particular when trying to assess an etiological diagnosis because of the absence of a full biological and radiological tests in our hospital, also because of the law economical and cultural level of our population. Also, the management of stroke in this age group needs not only our involvement but also that of the civil society, the government to build more physiotherapy, and speech rehabilitation dedicated centres. Finally, it is not a waste of time to insist another time on the major importance of prevention and the means of its development in our society.

## Conclusion

Our study gave us a lot of information, starting by the high incidence level of stroke among young people, the high rate of hypertension and embolic cardiopathies. Our hope is to share findings that will help the international community gathering data about stroke from all around the world to better understand it, and also, to convince our government of the urgent need to make more effort and energy into preventing this serious disease.
